# Risk factors for ulceration and amputation in patients with diabetic foot at risk: results form a tertiary care center

**DOI:** 10.1186/1758-5996-7-S1-A18

**Published:** 2015-11-11

**Authors:** Adriana Russo Fiore, Arnaldo Moura Neto, Karla Borges Daniel, Walter José Minicucci, Denise Engelbrecht Zantut Wittmann, Marcos Antonio Tambascia, Elizabeth João Pavin, Maria Cândida Ribeiro Parisi

**Affiliations:** 1 UNICAMP, Campinas, Brazil

## Background

Ulceration and amputation are severe complications of diabetes, leading to great morbidity and mortality. Of all lower limb amputations, about 50% are performed in these patients (1). In their lifetime, diabetics have a chance as high as 25% to develop a foot ulcer (2). After an amputation, mortality rates ranges from 13% to 40% at 1 year, 35% to 65% at 3 yrs., and 39% to 80% at 5 yrs. (3). The aim of this study was to assess the main risk factors of ulceration and amputation in patients with type 1 and type 2 diabetes.

## Materials and methods

A cross-sectional study was conducted in a tertiary hospital. Data was collected on the patients' first attendance in the neuropathic and diabetic foot unit, between June 2012 and September 2014. Statistical significance was set at 5%.

## Results

**Figure 1:**
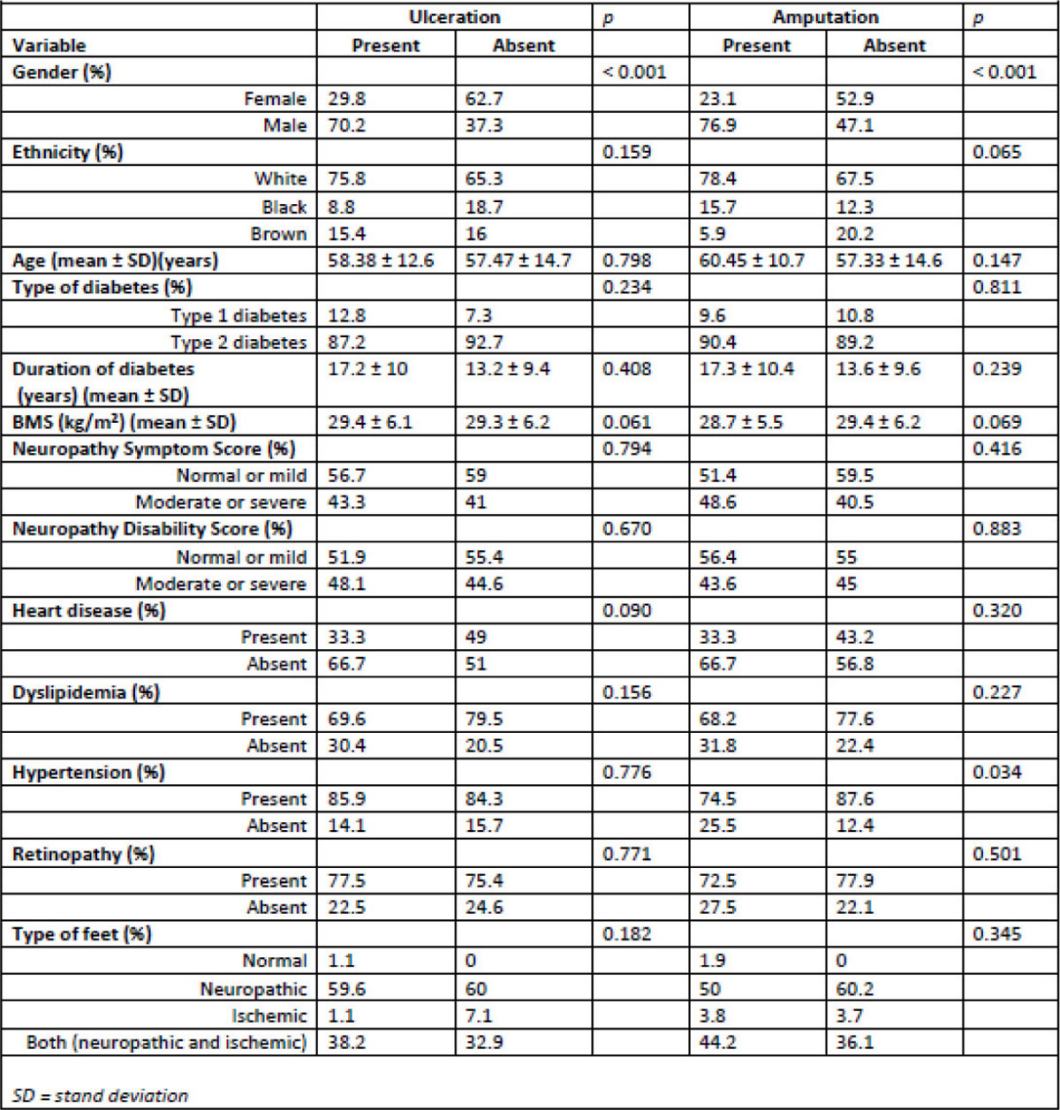
Associated factors with ulceration and amputation – description of the population (SD, standard deviation)

A total of 177 patients were evaluated. Ulceration and amputation were significantly more frequent in men (70.2% of all ulcerated patients and 76.9% of all amputees; p < 0.001). Hypertension was also a risk factor for amputation, present in 74.5% of amputated patients (p=0.034). In ulcerated patients, glycated hemoglobin was higher than in non-ulcerated patients (9.2±2% vs 8.35±1.99%; p=0.003). The independent risk factors for ulceration and amputation were neuropathic and neuro-ischemic foot at risk classification (OR 4.41; CI 1.83 – 10.65; p 0.001 and OR 1.21; CI 2.07 – 60.47; p=0.005, respectively), dyslipidemia increased (OR 9.2; CI 1.64 – 51.58; p=0.012and OR 5.68; CI 1.21 – 26.46; p=0.027, respectively) and microalbuminuria (OR 1.004; CI 1.001 – 1.006; p=0.011 and OR 1.005; CI 1.001 – 1.008; p < 0.001, respectively). There were no statistically significant differences between risk of ulcer or amputation and ethnicity, age, type of diabetes, duration of diabetes, BMI (body mass index), Neuropathic Symptom Score, Neuropathy Disability Score, heart disease and retinopathy (Figure 1).

## Conclusion

Besides neuropathic and neuro-ischemic foot, other factors such as hypertension, dyslipidemia and presence of microalbuminuria were variables associated with ulceration and amputation, supporting that treatment of the diabetic patient should also aim the control of cholesterol levels and microalbuminuria. Specific care and education should be directed to males, due worse outcomes. The knowledge of these risk factors is important for implementation of prevention strategies, avoiding future damage and disability.

